# Factors that Facilitate and Hinder the Comprehension of Patient Information Leaflets (PILs): A Brief Scoping Review

**DOI:** 10.3389/fphar.2021.740334

**Published:** 2021-11-10

**Authors:** Mariana Medina-Córdoba, Sara Cadavid, Andrés M. Pérez-Acosta, Valentina Amaya-Giraldo

**Affiliations:** School of Medicine and Health Sciences, Universidad del Rosario, Bogotá, Colombia

**Keywords:** comprehension, drug labeling, drug package insert, medicine package insert, patient information leaflet (PIL), readability, self medication, understanding

## Abstract

**Introduction:** Patient information leaflets (PILs) of medicinal products are informative documents that accompany medicines and explain their components, modes of use, interactions with other medicines, and other relevant issues. When patients do not adequately understand the information in the leaflets, they may engage in behaviors that affect their health (e.g., self-medication).

**Objective:** To identify patient-related factors and characteristics of PILs that can promote cognitive, emotional, and behavioral changes that lead to appropriate drug use practices. Additionally, we aimed to determine strategies that could be implemented to design leaflets that convey adequate information and are easier to understand.

**Method and Results:** We evaluated scientific articles published in databases and containing information on PILs suitability to be used in a patient population. A total of 51 articles were selected as the sample. Certain leaflet factors that favored or hindered understanding were identified (e.g., format in which the leaflets are presented, their structure, their adaptation to the sociodemographic and linguistic characteristics of the population, their wording…). Similarly, we also identified patient factors, such as previous experience taking the drugs referred to in the leaflet; the type of emotions experienced when reading the leaflets; the emphasis on the adverse effects of the medications; sociodemographic variables (i.e., age or educational level); and degree of interest in their own healthcare.

**Conclusion:** Patient and leaflet factors influence the comprehension of information in the PIL; hence, emphasis should be placed on these factors to increase treatment and medication adherence and to reduce health-risk behaviors.

## Introduction

There are numerous definitions of patient information leaflets (PILs); one refers to the documents provided to study participants, or their corresponding representatives, in clinical trials. As in other studies (Herber et al., 2014), in this study, PILs will be considered as the technical documents that contain written information about a drug and accompany it. In a PIL, the composition and conditions for usage of a drug are specified with the aim that patients can consume the drugs responsibly without incurring risks to their health (Vinker et al., 2007). PILs also include information on what precautions should be followed by the individual taking the drug, and the possible side effects that the drug may have. As a DeCS (Health Science Descriptors) term, PILs or Medicine Package Inserts are defined as “legal documents containing technical and scientific information and guidelines about medicines”, a definition that was set forth in some of the articles included in our study (Pizzol et al., 2019).

Given the importance of the PIL, it needs to be easy to comprehend and accessible so that anyone can understand it without difficulties. For this reason, PILs adapted to the characteristics of the target population have been developed. These documents facilitate understanding through the use of non-technical terminology, pictograms, and brief sections to try to answer any doubts that may arise when consuming the medication (Miquel et al., 2000). Additionally, although technological advances have increased considerably in recent years, users tend to consider the leaflet the primary source of information, even if it is confusing and hard to understand (Pizzol et al., 2019). Therefore, there is a need to review the practicality of these documents, ensure that they are relevant and frequently used, and use a psychological approach to explain consumer behavior.

When patients do not fully understand the information in the leaflets, they may engage in self-medicating behaviors (to see other factors that may lead to self-medication, see Bennadi, 2014). Some of these behaviors may be motivated or reinforced by the variability of the information provided by health institutions at the time of administering a drug (Clausen et al., 2016). This phenomenon shows the need to unify instructions for medication use between countries and/or regions. Therefore, it is necessary and relevant to study the characteristics of PILs to understand what makes them clear and effective when patients read them (Pizzol et al., 2019). When patients do not correctly understand or follow the instructions given in the PILs, there is a health risk that is not caused directly by the composition or the active principle of the drug itself but by its incorrect consumption. This, together with the lack of understandable information in the leaflets, has become a large-scale problem that affects the health of many, generates unforeseen expenses in the health system (Bologna, 2009), and affects the decision-making process regarding medication consumption (Clausen et al., 2016). Thus, there is a need to develop PILs that provide clear and precise information to protect people’s health.

This paper presents an overview of the recent literature (latest 15 years) that have identified factors facilitating and hindering the comprehension of PILs. Particularly, this mini scoping review primarily aims to describe the characteristics of a PIL that can promote cognitive, emotional, and behavioral change that leads to proper drug use practices. Additionally, we aim to identify the characteristics of the PIL that do not favor responsible and informed consumption of drugs. This way, it would be possible to suggest strategies that could be implemented in the future to design adequate and easily understandable leaflets. As stated before, the incorrect consumption of drugs is often influenced by variables that appear in the subject-leaflet interaction. Hence, this research focuses on studying higher psychological processes, such as memory, learning, understanding, and reasoning, as these components act as mediators between the PIL and the subsequent drug consumption behavior.

## Methods

### Search Strategy

The research question that guided this brief scoping review was addressed using a PIO (Population, Intervention and Outcome) format. Specifically, the population was anyone who was a user of medication, the intervention was the exposure to a PIL, and the outcome was the psychological interaction that occurred between the population and the intervention. We operationalized this psychological interaction in terms of the psychological factors that appear when people read PILs (i.e., readability, comprehension, learning, memory, reasoning, and impact, which referred to the consequences that reading the leaflet had on people’s decisions regarding medication). Thus, the outcomes of interest were the key mediating factors between the contact with the leaflet and the subsequent consumption behavior. Scopus, Pubmed, and Scielo were searched, and a total of 383 articles with the following key terms were found: patient AND patient information leaflet AND impact OR readability OR comprehen* OR learning OR memory OR reasoning. The information from the articles was organized in a database designed according to PRISMA specifications in order to apply the inclusion and exclusion criteria.

### Selection Criteria

We defined the following inclusion criteria: (I) Studies in which PILs were defined as “legal documents containing technical and scientific information and guidelines on drugs.” (II) Studies describing a specific target population of any age or nationality that has read the leaflets. (III) Studies in which the information in the leaflet was related to psychological variables that influenced people’s self-medicating behavior. (IV) Studies that included the terms memory, impact, readability, comprehension, learning, and/or reasoning. (V) Research written in English, Portuguese, French, or Spanish. The exclusion criteria were: (a) Studies describing other types of non-pharmacological inserts. (b) Studies written in languages other than those mentioned in the inclusion criteria, even if the abstract was translated into English. (c) Research published before 2005 (more than fifteen years prior to the beginning of the review). (d) Studies on pharmacological information strategies that included informative documents but not medical leaflets (e.g., documents provided before and after surgery or delivery).

### Selection Process

**FIGURE 1:**
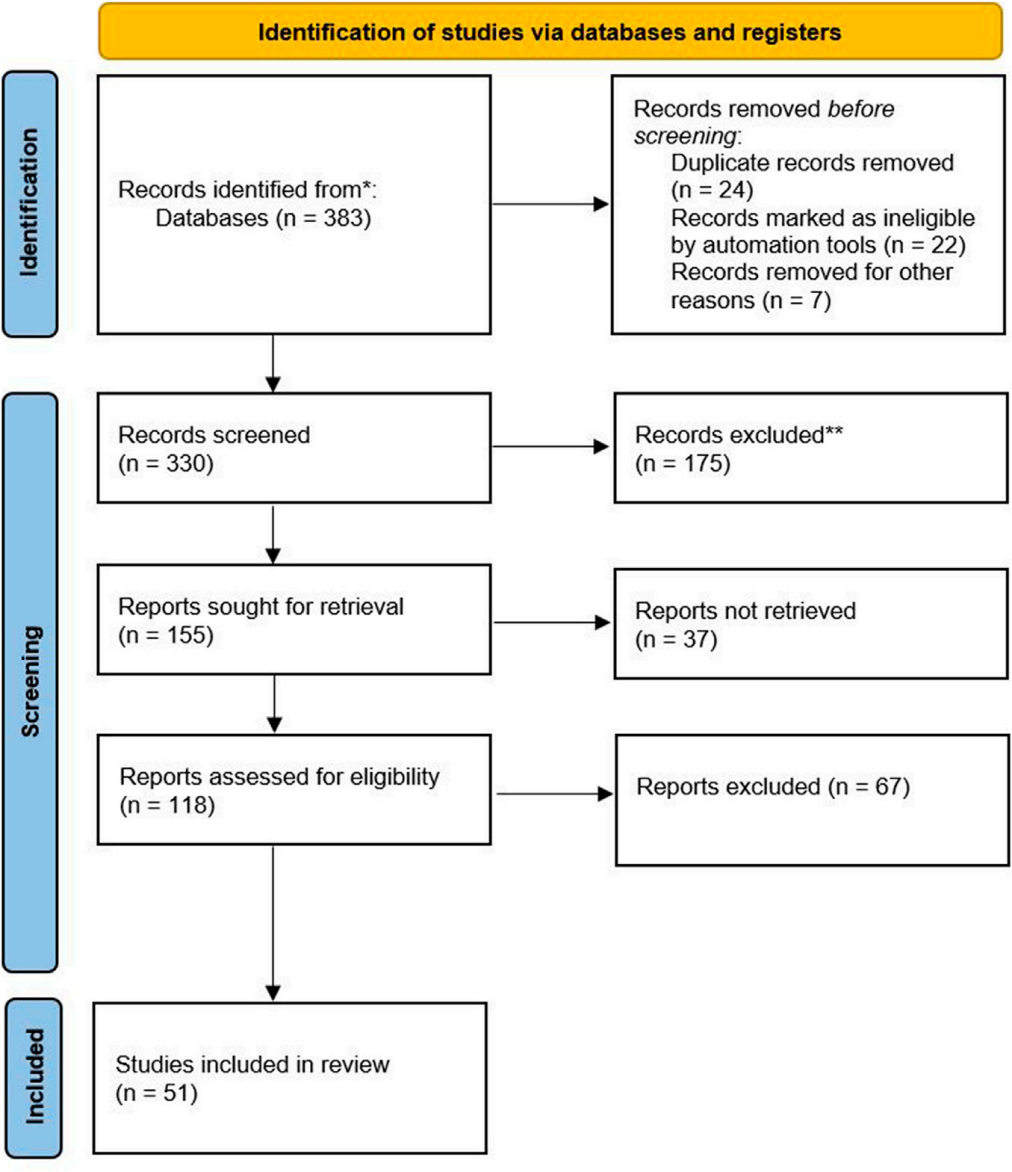
PRISMA 2020 flow diagram to represent the article selection process. Figure with yellow (identification of studies via databases and registers), blue (identification, screening and included) and white.

We obtained a total of 383 articles from the databases. These were organized according to a PRISMA-type classification. Two or three reviewers screened each record (title/abstract), leaving 155 publications preliminarily selected. Then, each report was screened by two reviewers, who identified 118 articles of interest. Disagreements between reviewers were solved by a discussion with the other two researchers. Then, scoping reviews, systematic reviews, briefing notes, and book chapters were excluded. Using the inclusion and exclusion criteria, the abstracts were reread and the sample size was narrowed down to 54 articles. Subsequently, the articles were reread, applying the selection criteria again and rechecking for duplicates, leaving 51 articles as the final number (see Figure 1). Since scoping reviews aim to provide an overview of the existing evidence regardless of the risk of bias, we did not assess the risk of bias of the articles included in this review (Tricco et al., 2018).

## Results

**FIGURE 2:**
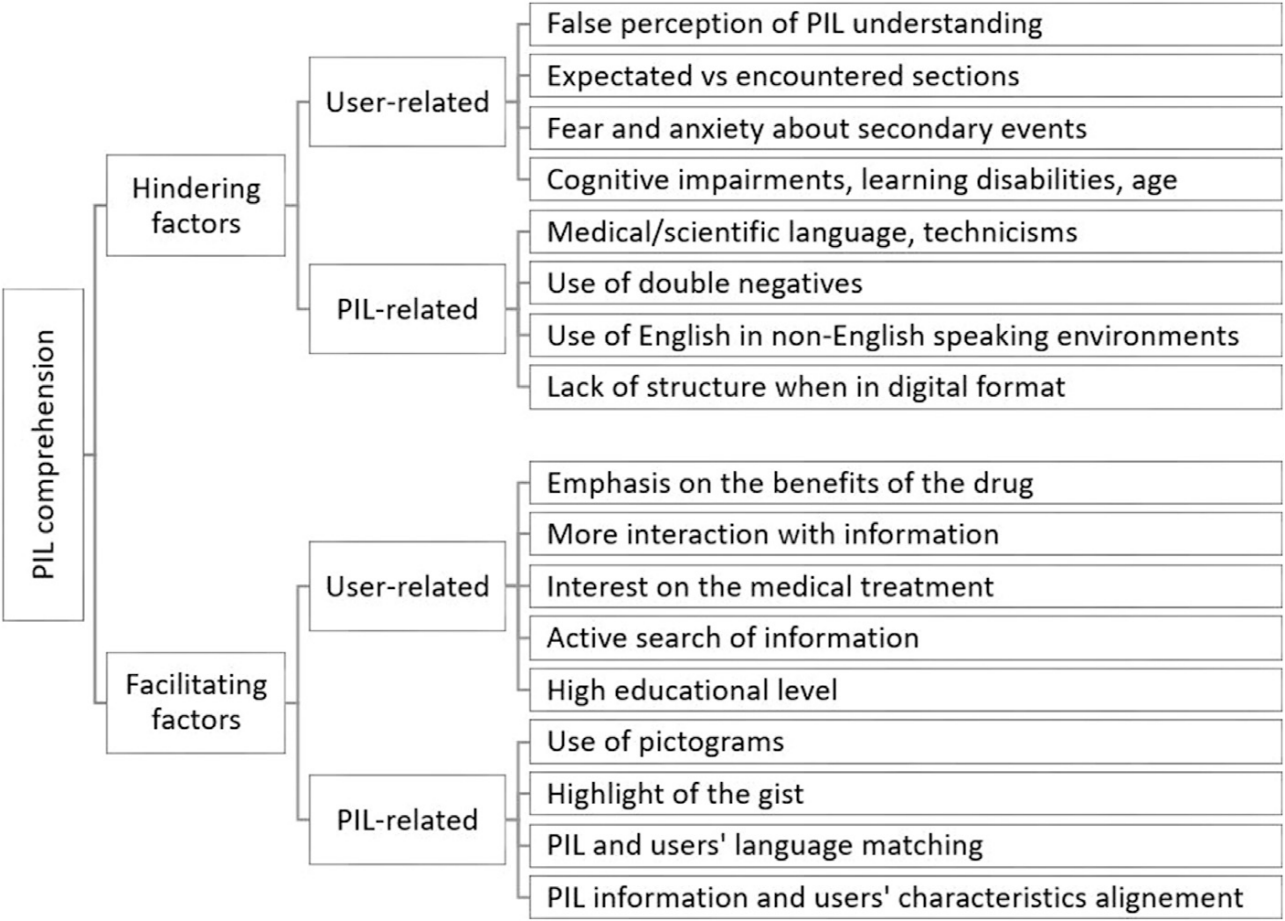
Aspects that facilitate and hinder PILs comprehension. Diagram where it can be read “PIL comprehension,” “Hindering fctors,” “Facilitating factors,” etc.

Results were obtained from 51 selected articles, whose countries of origin were Australia, Austria, Belgium, Brazil, Denmark, France, Germany, Ghana, India, Iran, Ireland, Malaysia, Netherlands, New Zealand, Norway, Pakistan, Qatar, Saudi Arabia, Slovenia, South Africa, Spain, Sweden, Tanzania, United Kingdom, and United States. Forty-nine articles were published in English, one in Spanish, and one in French. As the study’s main objective was to find the characteristics of the leaflets and users that aided or hindered the comprehension of the leaflet, the variables were divided into two categories: leaflet and user variables (see Figure 2). More information regarding each one of the articles included in the review can be found in the Supplementary Material of this paper.

### Leaflet variables

Some of the articles indicated the components of the PILs required to provide adequate information to consumers. These components included the names of the medicinal products, expected actions, appropriate forms of use, dosage, contraindications, external effects, special use for athletes, and storage conditions (Mwingira and Dowse, 2007). In addition, information regarding the possible side effects and interactions with other natural products and vitamins and the shelf-life of the active component is required in the leaflet (Ahmadi et al., 2019), and some leaflets have been reported as having information on the implications of its use and effects that the drugs would have in the elderly (Liu et al., 2014), infant-juvenile (Zidarič and Kreft, 2019), pregnant, and lactating population (Azari et al., 2018). Although there may be several sections within each package leaflet, patients consider that information regarding the usage instructions and side effects are more important than that on the composition and appearance of the medicinal products (Burgers et al., 2015).

The leaflets must be clear and concise in their explanations and must be available in the user’s language and adapted to their understanding (Clausen et al., 2016; Khodambashi et al., 2017). However, a considerable proportion of the leaflets evaluated in these articles did not meet these characteristics as they were not understood by the general public (Rajasundaram et al., 2006; Symonds et al., 2011; Brooke et al., 2013; Spinillo, 2014; Bennin and Rother, 2015; Alaqeel and al Obaidi, 2017; Haller et al., 2019), nor did they meet the parameters of utility (Sukkari et al., 2012), readability (Kasesnik and Kline, 2011), adaptation to the educational level of the target population (Cronin et al., 2011), or use of non-medical terminology (Hirsh et al., 2009; Bennin and Rother, 2015). Participants in other studies confirmed that the leaflets were clear, legible (Gustafsson et al., 2005; Williamson and Martin, 2010; Potter et al., 2014), and useful (Edwards et al., 2013), even though the participants showed low scores in a test on knowledge acquired from the PILs (Alaqeel and al Obaidi, 2017). Additionally, there is also evidence that only some sections were difficult to understand (Gustafsson et al., 2005). Thus, studies examining the PILs’ clarity showed mixed results.

The information format presentation was found to play a relevant role to promote appropriate PIL usage. In five out of the 51 articles included in this review, researchers explored the effect of pictograms on PIL comprehension and found that pictograms facilitated the PIL understanding (Mansoor and Dowse, 2006; Mwingira and Dowse, 2007; Dowse et al., 2014; Spinillo, 2014; Spinillo, 2016). The combined format of the text and pictograms produces robust and useful representations of the information, which leads to easier decision-making for both professionals (Arsalan et al., 2015) and patients (Mansoor and Dowse, 2006; Dowse et al., 2014; Hammar et al., 2016). The use of section titles is another format issue that seems useful for a faster and more comprehensible search of the information in the PIL (Dickinson et al., 2016), as sometimes patients report having problems finding the information (Pander Maat and Lentz, 2010; Pander Maat et al., 2015). Also, PILs on a physical format have shown to be more structured in their content and explanation than PILs on a digital format (Afreh et al., 2017). In other study, the PILs approved by the national health authorities proved to be more readable and understandable than the information available on the internet (Mira et al., 2013), even though the PILs did not meet the expected quality criteria. The studies examining patients’ adherence to treatment related to PILs’ characteristics showed that the use of grammatical negatives hindered comprehension (Burgers et al., 2015). Poor readability and comprehensibility affected the patients’ behavior, leading to a lower degree of medication adherence (Burgers et al., 2015; van Beusekom et al., 2016; Munsour et al., 2017). In contrast, pictograms in PILs seemed to be beneficial to increase patients’ adherence (Mansoor and Dowse, 2006). Adherence also increased in a study where PILs were administered together with clinical pharmacists patient-education interventions (Ashok et al., 2017).

### User variables

Several of the reviewed articles indicated that the participants’ perception of understanding was higher than their real comprehension. For instance, participants could not reproduce what they read in the PIL in their own words (Mwingira and Dowse, 2007). Additionally, participants also did not perform well in questionnaires regarding their knowledge about PILs, especially in sections on contraindications (Gustafsson et al., 2005; Alaqeel and al Obaidi, 2017) and risks of interactions (Gustafsson et al., 2005). Likewise, the findings highlight that the sections of the PILs do not match patients’ expectations regarding the importance of the contents. For example, patients would rather know the benefits and risks of taking the drug than know its composition (Maat and Lentz, 2011). To know how they can feel better, users also prefer to know the benefits of the drug rather than their side effects (Hirsh et al., 2009).

Although it is difficult to understand the leaflets, patients showed a great interest in learning about medicinal products (Hirsh et al., 2009), so they tended to search the internet for some prototypes of virtual leaflets that provided them with information easily and quickly (Afreh et al., 2017; Dickinson et al., 2017; Ahmadi et al., 2019). Further, the advice of a health professional that complements the information in the leaflets increases adherence to treatment (Ashok et al., 2017) and the active search for information (Symonds et al., 2011; Potter et al., 2014). In addition, people’s opinion of the leaflets are based, above all, on the healthcare professionals’ recommendations on the use of medicinal products and their benefits, main characteristics (Kohler et al., 2009), and side effects (Schmitz et al., 2017).

Previous experience plays an important role in how the PIL is used. In fact, the main reason parents medicate their children seems to be their own experience with the symptoms that the minors present with (Afreh et al., 2017). However, reading PILs significantly increased knowledge about the medicinal product despite not having much background information before reading it (Dowse et al., 2014). Also, when people read PILs, the information they store is combined with or framed by preexisting mental representations that people have previously formed about medicines (Kohler et al., 2009). People who present with side effects after taking the drugs tend to reduce the perception of causality between drug use and said symptom if the leaflet presents the information through affirmative sentences rather than negative ones (Webster et al., 2018). In addition, side-effects expectations prior to ingestion lead to the belief that the common or very common side effects and adverse effects proposed in the leaflets have a higher incidence than they actually do (Webster et al., 2017a). This may be related to an overestimation of side or adverse effects, making people less willing to consume the medicinal products (Webster et al., 2017b). Thus, some people sometimes understand medication instructions differently than the average population. This could happen when the information is ambiguous (Spinillo, 2016) and is expressed using scientific terms instead of plain language (Rajasundaram et al., 2006; Hirsh et al., 2009; Bennin and Rother, 2015). Additionally, it has been found that the greater the variety of medications ingested by a patient, the less is the understanding of the PILs (Gupta et al., 2005).

Unpleasant emotions play an important role in the use of PIL, both for direct users and for minors who are administered the medicinal product by their parents, as anxiety precipitates the decision to ingest or administer the drug without prior consultation with a professional or reading of the PIL (Afreh et al., 2017). The information presented in the leaflets can generate emotions, such as anxiety about ingestion, which can cause a change in the way of taking the drugs (i.e., increasing or decreasing doses, or discontinuing medication, or taking medications that are at home or that have worked for another person in the past without consulting professionals) (Thomas et al., 2018). Reading the leaflet can reduce the drug intake due to increased knowledge regarding side effects (Schmitz et al., 2017). PILs reading can trigger anxiety and fear, although no quantitatively measurable significant variation has been found in terms of these emotional reactions (Herber et al., 2014). Additionally, patients may resort to reading the leaflet driven by the need to know if something new or different will happen to them after the intake (Krska and Morecroft, 2013).

In terms of sociodemographic variables, information-seeking behavior differs between sexes as women, compared with men, tend to search for more information (Dickinson et al., 2017). Also, natural aging appears to increase cognitive storage and processing of the leaflet’s general idea, rather than the specific details. Therefore, adults find it easy to understand the leaflets that present the information in a combined textual–pictographic format (Dowse et al., 2014). In fact, evidence was found regarding the existence of certain groups of the population that would face difficulties in reading and understanding the PIL, such as people with learning difficulties (Young et al., 2018) and the population with some type of cognitive (Pertl et al., 2014) or visual impairment, like older adults (Geest et al., 2005). In contrast, people with a higher degree of literacy tend to better understand the importance of images in leaflets (van Beusekom et al., 2016) and the information in the leaflet itself as the leaflets are developed for people with a medium educational level, such as those who have passed grades six through ten (Williamson and Martin, 2010; Brosnan et al., 2012; Arsalan et al., 2015). However, people with a higher educational level, younger age, and higher socioeconomic status are most likely to self-administer the medicinal product (Pander Maat et al., 2015).

## Discussion

This study aimed to identify the cognitive, behavioral, and emotional factors that facilitate or hinder the acquisition of information from PILs by patients who buy the drugs. The findings show that patients are aware that PILs are necessary for understanding the drug. However, in terms of PILs’ general public acceptance, results are not homogeneous. PILs are perceived as useful, but they can also generate adverse emotional reactions (Herber et al., 2014; Afreh et al., 2017; Thomas et al., 2018). In addition, the PILs in the current market are considerably illegible (Rajasundaram et al., 2006; Symonds et al., 2011; Brooke et al., 2013; Spinillo, 2014; Bennin and Rother, 2015; Clausen et al., 2016; Alaqeel and al Obaidi, 2017; Haller et al., 2019; Zidarič and Kreft, 2019), impractical to use (Sukkari et al., 2012), and can generate emotional discomfort and confusion due to their format. Therefore, several factors that facilitate the PIL understanding need to be consolidated in a future proposal to improve the PILs.

First of all, it would be important to organize PIL sections according to the most immediate needs of the users. This way, patients’ expectations about the information location and the place where they look for information in the PIL would match. Specifically, patients expect to find information in the leaflets in the following order: benefits, side effects, and contraindications (Hirsh et al., 2009; Burgers et al., 2015). In addition, they should be presented in a typographic–pictorial format with images that allow a better understanding of the PIL. If possible, PILs should allow a certain degree of active interaction with the information to create a more lasting memory footprint (Dowse et al., 2014; Spinillo, 2014; van Beusekom et al., 2016). Likewise, although physical formats are recommended, as already described, virtual formats could be useful to complement the information and create a broader didactic spectrum.

Secondly, according to the results presented (Hirsh et al., 2009; Cronin et al., 2011; Bennin and Rother, 2015), the PIL should be written in a simple language that uses general terms that are as non-scientific as possible as many of the consumers could have a low educational level. In addition, the PIL should be adapted to the target population using a larger typographic style and more straightforward terms with a focus on the population that finds it difficult to read the PILs, such as the elderly population and those with cognitive and/or learning difficulties (Geest et al., 2005; Pertl et al., 2014; Young et al., 2018).

Finally, a trained healthcare professional could guide patients after a detailed reading of the PIL to solve any doubts that may arise (Ashok et al., 2017). This guidance could be an effective strategy to minimize the overestimation of the risk associated with the intake, reduce users’ overestimation of their own understanding of the leaflet, emphasize the benefits of treatment, and answer personalized questions about the interactions between various medications or other substances and the drug in question. Thus, patient adherence to the treatment would be increased, and the risk of inappropriate self-medication leading to poor health could be reduced.

In sum, we presented a first approach to studying an overriding subject in the healthcare context. We hope that our work help to raise researchers’ interest in this particular area, which in turn could lead to an increase in the number of studies focused on improving PILs as defined in the introduction (technical documents that contain written information about a drug and accompany it). Research has found some PIL-related variables that could be easily implemented to reduce risks associated with medication consumption errors. Therefore, PILs designers are called to explore further and use these variables to protect people’s health.

As limitations of our work, we identified that the studies are difficult to compare with each other due to the great diversity of methods used to carry out the research. Research could also be extended to other types of patient information leaflets, such as those found in clinical trials or before a surgical procedure. In this sense, our review would constitute a first step towards identifying factors that are decisive for the improvement of PILs. Much more research is needed, and further systematic reviews or meta-analyses on the subject would be warranted.
